# Cognitive aid use improves transition of care by graduating medical students during a simulated crisis

**DOI:** 10.3402/meo.v21.32118

**Published:** 2016-07-18

**Authors:** Brooke Bauer, Annette Rebel, Amy Dilorenzo, Randall M. Schell, Jeremy S. Dority, Faith Lukens, Paul A. Sloan

**Affiliations:** Department of Anesthesiology, University of Kentucky, Lexington, KY, USA

**Keywords:** cognitive aid, transition of care, simulation, crisis management, rapid response, communication

## Abstract

**Background:**

Residents are expected to have transition of care (ToC) skills upon entering graduate medical education. It is unclear whether experience and training during medical school is adequate.

**Objective:**

The aim of the project was to assess: 1) graduating medical students’ ability to perform ToC in a crisis situation, and 2) whether using a cognitive aid improves the ToC quality.

**Methods:**

The authors developed simulation scenarios for rapid response teams and a cognitive aid to assist in the ToC during crisis situations. Graduating medical students were enrolled and randomly divided into teams of three students, randomly assigned into one of two groups: teams using a cognitive aid for ToC (CA), or not using a cognitive aid (nCA). In the scenario, teams respond to a deteriorating patient and then transfer care to the next provider after stabilization. Three faculty reviewed the recording to assess completeness of the ToC and the overall quality. A completeness score was expressed as a fraction of the maximum score. Statistical analysis was performed using a *t*-test and Mann-Whitney U test.

**Results:**

A total of 112 senior medical students participated: CA *n*=19, nCA *n*=17. The completeness score of the ToC and overall quality improved when using the cognitive aid (completeness score: CA 0.80±0.06 vs. nCA 0.52±0.07, *p*<0.01; ToC quality: CA 3.16±0.65 vs. nCA 1.92±0.56, *p*<0.01). Participants’ rating of knowledge and comfort with the ToC process increased after the simulation.

**Conclusion:**

The completeness of information transfer during the ToC process by graduating medical students improved by using a cognitive aid in a simulated patient crisis.

The transfer of patient care from one provider to another is referred to as the transition of care (ToC) process. Multiple factors have increased the need for ToC between healthcare providers, including a restriction of duty hours for residents, transfer of patients between hospitals for specialist care, and the transfer of patients among specialized teams within a hospital (1). The Joint Commission has identified communication failures during this handover process as a major contributor to medical errors (2). Moreover, communication failures occur more frequently when ToC occurs during times of patient crisis (3–6). A recent study assessed the quality of the ToC process by anesthesiology residents in a crisis situation and found that there was a significant loss of information during the ToC process (7, 8).

The ToC process is a critical component of safe patient care (5). However, the medical school curriculum may not provide structured training and documentation of competency in the ToC process. Informal and observational training of handoffs during clinical training may be the only way ToC skills are learned. It is unclear if this clinical experience and training are sufficient to prepare graduating medical students adequately for ToC during clinical rotations where it is more focused on census based handoff during shift change and more specifically, ToC in crisis situations.

We hypothesized that the ToC training that medical students receive during medical school is not sufficient for adequate ToC during crisis situations, resulting in the potential for significant information loss during the process. The use of a cognitive aid may improve the completeness of information transfer during the ToC process by providing structure and organization.

The aim of this project was to assess: 1) graduating fourth-year medical students’ ability to perform a ToC process in a crisis situation, and 2) whether the use of a cognitive aid improves the quality of the ToC process of these graduating medical students in crisis situations in order to prepare them for this skill entering residency.

## Methods

Following institutional review board approval and informed student consent, graduating fourth-year medical students from the University of Kentucky (UK) (*n*=112) agreed to participate in this project. The simulation scenario was conducted 2 weeks prior to graduation as a part of a weeklong ‘intern preparation’ course designed to prepare the graduates for the upcoming challenges of their intern year (9).

### Simulation scenario

The authors developed, by consensus, two different simulation scenarios. The scenarios, similar in complexity but not identical, were assigned randomly between the two groups of students (using a cognitive aid [CA] and without cognitive aid [nCA]). The medical students were randomly divided into teams of three, thus forming a rapid response team (RRT) with self-assigned roles (team leader, respiratory therapist, and ICU nurse). Before the scenario, all medical students received formal instruction in ToC by watching a 15 min instructional video produced by the authors, containing a brief review of the ToC process. The video, a ‘just-in-time’ teaching method (instruction immediately prior to application) (10), covered knowledge components about the ToC process and also explained the different components of the handoff, including structured methods for obtaining and relaying patient information. The training video included a demonstration of a correctly performed patient care handoff. After watching the video, the students entered the simulation room. The patient simulation used a computer-controlled mannequin (Laerdal Sim Man 3G™). The scenario began with the RRT arriving at the bedside of a deteriorating patient. The bedside nurse (confederate 1) was present and provided baseline patient information. The student team leader initiated communication with the bedside nurse, obtained the necessary information, and delegated tasks to be performed by the other team members. When the team was satisfied with the amount of information received, the retrieving information portion was concluded and a read back from the confederate was performed. The read back repeated all the pertinent information about the patient and situation in a structured format to the RRT, therefore ensuring that all student teams received complete and identical information.

**Fig. 1:**
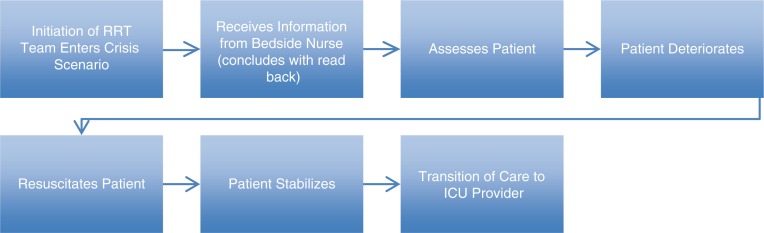
Scenario outline. This figure depicts the scenario outline for the medical students involved in the transition of care simulation. The medical students are split into teams of three to form a rapid response team (RRT) prior to entering the scenario. The bedside nurse then gives the team information about the deteriorating patient. The RRT assesses the patient who then deteriorates, requiring the RRT to resuscitate the patient. After the patient stabilizes, the RRT performs transition of care to the ICU provider.

In the second stage of the scenario, the patient deteriorated into cardiac arrest, and the RRT was required to perform Advanced Cardiac Life Support (ACLS). Following CPR, patient intubation, and intravenous epinephrine, the patient's medical condition stabilized. Confederate 2 (‘ICU fellow’) then entered the scenarioand received report from the medical student team. The scenario outline is described in Figure 1. Directly after conclusion of the scenario, all participants received feedback and a debriefing session by the confederates and the simulation instructor. The complete scenario was videotaped with voice recording for data analysis.

### Cognitive aid

**Fig. 2:**
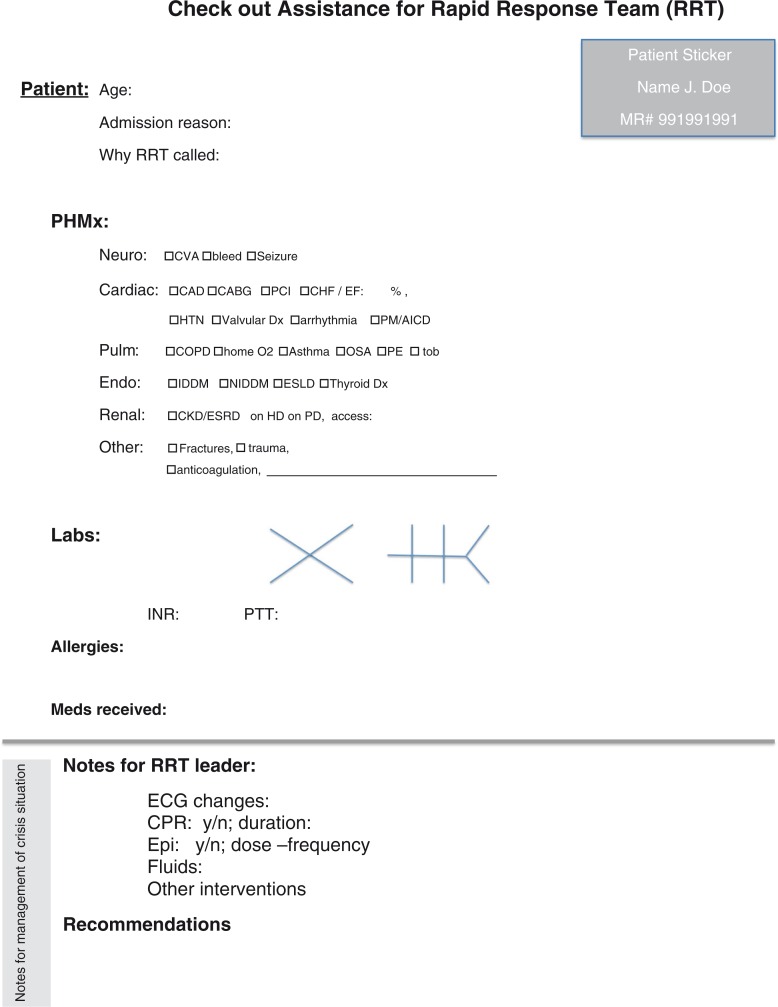
Cognitive aid. To assist in the ToC process, the group of medical students (Group CA) were offered the cognitive aid in the figure.

The paper-based cognitive aid was developed by faculty consensus and has been validated previously (8, 11). Prior to the simulation scenario, the student teams were randomly assigned to the following groups: ToC with cognitive aid (CA) or ToC without cognitive aid (nCA). In group CA, the cognitive aid was provided for the team leader upon entrance into the simulation room (Fig. 2).

### Evaluation

Following conclusion of the scenario, expert faculty (*n*=3), blinded to the student group assignment, reviewed voice recordings from the video to maintain the integrity of the blinding process. The completeness of the report was graded using a scenario-specific checklist. The completeness score was reported as a fraction of complete and correct information that was transferred to the ICU provider. In addition to assessing the completeness of the ToC process, expert faculty summarized the overall quality of the ToC process in a subjective manner using a modified Likert scale scoring tool (1–5; 1=unsatisfactory; 5=outstanding).

### Surveys

**Fig. 3:**
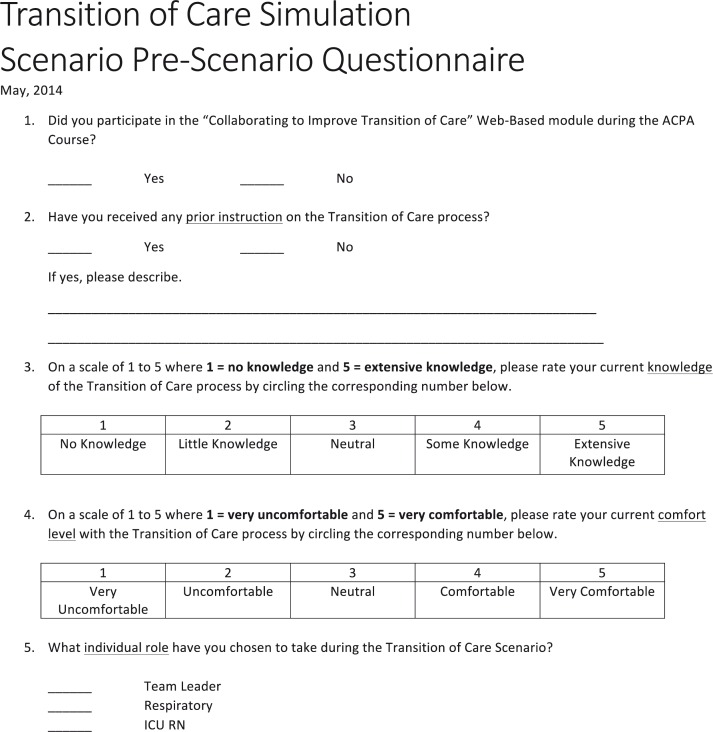
Pre-scenario survey. Prior to watching the instructional video and completing the simulation scenario, the participating medical students were asked to complete the pre-scenario survey in the figure.

**Fig. 4:**
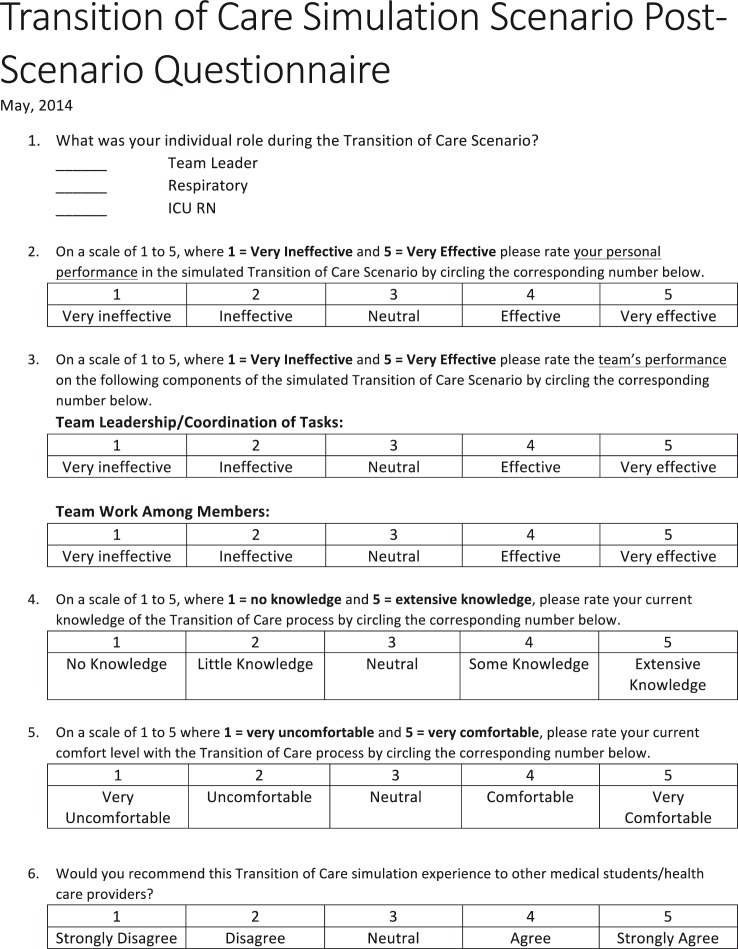
Post-scenario survey. The medical students rated their performance and comfort level with the ToC process with the post-scenario survey after viewing the instructional video and participating in the simulation scenario.

Prior to the instructional video and the simulation scenario, all participating medical students were asked to assess their individual knowledge (1–5; 1=no knowledge; 5=extensive knowledge) and comfort level (1–5; 1=very uncomfortable; 5=very comfortable) with the ToC process. The post-scenario survey included a similar self-reflective modified Likert-based rating of individual and team performance in addition to an assessment of the learning experience pertaining to their knowledge and comfort level after participating in the simulation (Figs. 3 and 4).

### Statistical analysis

All scores are reported as mean±SD for each group. Statistical analysis of all data was performed using an unpaired *t*-test and Mann-Whitney test, since normal distribution of parameters is not known, with a statistical significance set at *p*<0.05 for both tests.

## Results

A total of 112 senior medical students participated in the study. After random team configuration and group assignment, 19 teams completed the simulation using the cognitive aid (CA *n*=19) and 19 teams did not use the cognitive aid (nCA *n*=19). Two teams from the nCA group were excluded because of incomplete data collection due to microphone malfunction during the recordings (nCA *n*=17). Two teams (one in each group) consisted of only two participants because the third medical student was not available. In these teams, the role of the ICU nurse was eliminated and confederate 1 assisted during the ACLS portion of the scenario.

The assessment of the completeness and efficiency of the ToC process using delayed analysis by reviewing video recordings resulted in the completeness score. Without the use of a cognitive aid, substantial amounts of information were lost during the ToC process from one provider to another (Table 1).

**Table 1:** Completeness and overall ToC quality

	Group nCA	Group CA	
		
	*N*=17	*N*=19	*p*
Completeness score	0.52±0.07	0.80±0.06*	<0.01
Overall ToC quality score	1.92±0.56	3.16±0.65*	<0.01

The table presents the completeness score of the ToC process, assessed using scenario-specific checklists. The overall ToC quality was assessed using a modified Likert scale–based scoring tool (1=unsatisfactory to 5=outstanding). Data are shown as mean±SD (nCA=no cognitive aid; CA=with cognitive aid).

* *p*<0.05.

The use of a cognitive aid significantly improved the completeness score of the ToC process (CA 0.80±0.06 vs. nCA 0.52±0.07, *p*<0.01). The overall ToC process ratings also benefitted from the use of the cognitive aid (CA 3.16±0.65 vs. nCA 1.92±0.56, *p*<0.01).

### Survey data

**Fig. 5:**
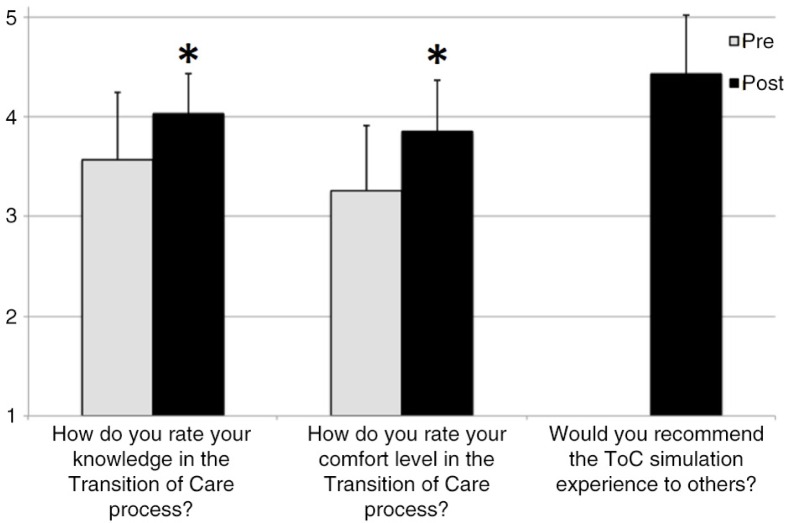
Pre- and post-scenario survey results. The figure illustrates the results of the pre-scenario survey (*n*=110) and post-scenario survey (*n*=107). The participants rated their knowledge and comfort level concerning the ToC process before (pre, gray) and after (post, black) the simulation experience on a modified Likert scale (1=no knowledge/very uncomfortable to 5=extensive knowledge/very comfortable). The participates were asked if they would recommend the scenario experience to others (1=very unlikely, 5=very likely). The data are shown as mean±SD. *p<0.05.

One hundred ten students returned the pre-scenario survey (110/112; 98%), and 107 students returned the post-scenario survey (107/112; 96%). The pre-scenario survey indicated that 57 participants (52%) had some form of prior instruction in the ToC process. Despite prior training, knowledge and comfort level with the ToC process was rated as only neutral (Fig. 5). Following the simulation experience, the post-scenario survey indicated an improvement of ToC knowledge (pre 3.57±0.67 to post 4.03±0.40, *p*<0.01) and ToC comfort level (pre 3.26±0.65 to post 3.85±0.51, *p*<0.01). The medical students indicated post-simulation that they would recommend this ToC experience to others (4.43±0.59 on a 5-point modified Likert scale [1=very unlikely; 5=very likely]).

## Discussion

The ToC process may not be included in some medical school curriculum and is rarely practiced during clinical rotations since medical students are not commonly responsible for the ToC of a patient to another provider. However, it becomes a daily requirement in almost all specialties in graduate medical education and has gained attention with the limitations on duty hours (12) and the possibility of increased loss of information with more frequent ToC. It seems imperative to introduce the concept to medical students during their medical school curriculum and allow them to begin practicing ToC prior to graduation. The Accreditation Council for Graduate Medical Education has even emphasized the need to provide more structural ToC training during residency, and ToC is one of the six focus area of the ACGME CLER program (13). Medical students close to graduation should be prepared to take on junior resident responsibilities and should be competent to perform ToC in all clinical situations (14).

Efficient and complete ToC becomes essential during critical events necessitating an escalation of care (6). Proficiency in ToC is critical at the beginning of residency training (5). Therefore, we chose to assess ToC ability in all graduating medical students and to explore possible improvement opportunities for education in this skill including the use of a cognitive aid to improve ToC completeness (8).

The main findings of our study are as follows: 1) graduating medical students with previous informal clinical training and video-based training performed the ToC process with a significant loss of information, 2) simulation-based ToC experience increased the subjective knowledge and comfort level of graduating medical students with the ToC process in crisis situations, and 3) the use of a cognitive aid improves the ToC completeness.

The current training medical students receive in medical school for ToC may not be sufficient. In our pre-scenario survey, approximately one-half of the participants indicated they did not receive prior ToC teaching. ToC instruction may be more commonly focused on ‘census based handoff’ at shift transition, but not addressing the needs if information transfer must be prioritized due to time constraints (15). At our institution, the medical school curriculum provides formal ToC training using web-based podcasts in addition to the informal handoff training during their clinical clerkships. Due to integration of the majority of ToC training into their clinical work and the small amount of structured ToC training, the medical students may actually perceive that they have not received any training in patient care handoff at all. It explains the perceived lack of knowledge and confidence found during the pre-scenario survey. Other medical colleges have ToC training during a specific clerkship or during the third year (16, 17). Rarely, simulation-based training is offered to teach and assess ToC competency. However, simulation provides an optimal learning environment for instruction and evaluation of the ToC process (7, 8, 18). As a response to our project and to the obvious need to prepare our medical students for clinical practice, our medical school curriculum has been reorganized to emphasize structured ToC training using simulation and standardized patients.

Our study suggests the feasibility of providing a safe learning environment for ToC in crisis situations by using the simulation scenario. To improve the quality of the process, we constructed a specific cognitive aid to assist in ToC during time-sensitive situations. Our project was able to confirm the positive impact of a cognitive aid on the ToC process. Other studies have supported the value of a cognitive aid for transfer of important information (19–23).

A delayed assessment (video review) for completeness and information organization indicated a statistically significant difference when a cognitive aid is utilized in the ToC presentation.

The use of a cognitive aid to help with the ToC process is useful and could be introduced during medical school. With multiple studies from healthcare and aviation industry supporting use of a cognitive aid, it seems plausible that one could choose most any of the formats and achieve an improvement in organization and completeness of handover (24–27). A recent review of healthcare handoff practices showed that the majority of published evaluation studies used departmental and specialty specific handoff tools (28). Further development of the cognitive aid format for the ToC process is needed, and further multi-institutional research is planned in this direction. The trend in handoff-related publications indicates a preference for electronic handoff tools with possible electronic medical record (EMR)-integrated information population (28). However, we are not aware of any study showing superiority of one format over another. Currently, the reasons for choosing one format over another should be based on equipment availability, system-based practice, and provider preferences (28).

There are several limitations to our project. In addition to the single-center design and limited sample size, the format of the cognitive aid limits our conclusions. Although a separate study was conducted to determine the most beneficial cognitive aid design, that study may also present limitations due to bias (11). It is possible that the developers of the aid were more comfortable with a paper/hand-written aid, while the graduating medical school population may obtain more benefit from an electronic form of the document (25, 28, 29). Non-familiarity of the cognitive aid by the medical students could also be a limitation to our study since the cognitive aid is not a routine tool used in standard clinical practice at our hospital.

In conclusion, our single-center simulation-based study indicated that the use of a cognitive aid during a high-acuity patient handover situation is beneficial in relaying correct, complete, and concise information to the next patient care provider. Further studies are needed to determine the best method(s) of preparing medical students to perform skilled ToC during graduate medical education. When a patient is transferred between levels of care, the use of a cognitive aid should become standard practice.
